# PixelCraftSR: Efficient Super-Resolution with Multi-Agent Reinforcement for Edge Devices

**DOI:** 10.3390/s25072242

**Published:** 2025-04-02

**Authors:** M. J. Aashik Rasool, Shabir Ahmed, S. M. A. Sharif, Mardieva Sevara, Taeg Keun Whangbo

**Affiliations:** 1 Department of IT Convergence Engineering, Gachon University, Sujeong-Gu, Seongnam-si 461-701, Gyeonggi-do, Republic of Korea; aashikrasool@gachon.ac.kr (M.J.A.R.); shabir@gachon.ac.kr (S.A.); sevara1998@gachon.ac.kr (M.S.); 2 Opt-AI Inc., LG Sciencepark, Seoul 07520, Republic of Korea; sharif@opt-ai.kr; 3 Center of Artificial Intelligence for Medical Instruments, Incheon 21982, Republic of Korea

**Keywords:** image super-resolution, lightweight image super-resolution, internet of things, reinforcement learning, computer vision

## Abstract

Single-image super-resolution imaging methods are increasingly being employed owing to their immense applicability in numerous domains, such as medical imaging, display manufacturing, and digital zooming. Despite their widespread usability, the existing learning-based super-resolution (SR) methods are computationally expensive and inefficient for resource-constrained IoT devices. In this study, we propose a lightweight model based on a multi-agent reinforcement-learning approach that employs multiple agents at the pixel level to construct super-resolution images by following the asynchronous actor–critic policy. The agents iteratively select a predefined set of actions to be executed within five time steps based on the new image state, followed by the action that maximizes the cumulative reward. We thoroughly evaluate and compare our proposed method with existing super-resolution methods. Experimental results illustrate that the proposed method can outperform the existing models in both qualitative and quantitative scores despite having significantly less computational complexity. The practicability of the proposed method is confirmed further by evaluating it on numerous IoT platforms, including edge devices.

## 1. Introduction

Single image super-resolution (SISR) refers to the reconstruction of a high-resolution (HR) image from its corresponding low-resolution (LR) image counterparts [1,2,3,4]. This is a fundamental task in computer vision with a broad spectrum of practical applications [5,6,7]. Contemporary deep-learning-based super-resolution (SR) approaches have consistently demonstrated significant results in enhancing the quality of reconstructed HR images [8,9,10]. Nevertheless, these approaches have a higher computational cost and memory requirement, rendering them impractical for deployment on constrained resource devices [11,12,13]. Hence, enhancing the efficiency of SISR approaches and developing lightweight models that can generate superior HR images at lower computational costs is crucial [14,15]. This task is specifically called the efficient super-resolution (ESR) task [16].

Recently, learning-based image translation methods have demonstrated substantial domination in the construction of SR images. Initially, Dong et al. [17] introduced an approach called super-resolution convolutional neural network (SRCNN), which utilizes a three-layer-based network and reformulates a sparse-coding approach. However, this convolutional neural network (CNN) has proven ineffective and is often prone to producing artifacts in the generated SR images owing to shallow convolution with a small receptive field. Subsequently, VDSR [18], FSRCNN [19], and ESPCN [20] were introduced to address this issue. However, even though these methods considerably reduce model complexity, they also reduce model accuracy, rendering them inviable for sensitive domains such as telemedicine and medical imaging [21,22].

Considering the significance of both the model accuracy and complexity, the NTIRE challenge for lightweight SR was introduced to propose models with the lowest possible compromise in accuracy while reducing complexity. The RFDN [23] approach demonstrated a notable improvement in ESR. In this approach, the authors convert high-level features from an HR image into an LR equivalent. The RFDN aims to extract and transfer feature representations from a deep, complicated teacher network to a shallower student network. This procedure reduces processing costs and improves the interpretability and generalization abilities of the model. Nevertheless, the performance of an RFDN is intricately tied to the quality and robustness of the teacher model, from which it extracts characteristics. If the teacher model is inaccurate or biased, then these issues can be transferred to the student model. To address this issue, Kong et al. [1] proposed an RLFN architecture that learns residual local features by employing three convolutional layers. However, training an RLFN can be challenging, primarily because it is a recurrent network, and such networks are particularly prone to overfitting.

Nevertheless, these models significantly reduced the number of model parameters and memory while maintaining a reasonable threshold of model accuracy. However, complex image sets remain shallow and sometimes produce suboptimal results. Additionally, these models struggle to generalize across diverse image contents and demand substantial data for training, making them impractical in specific fields such as the healthcare domain, in which data acquisition from patients requires ethical considerations and consensus from various authorities.

**Figure 1:**
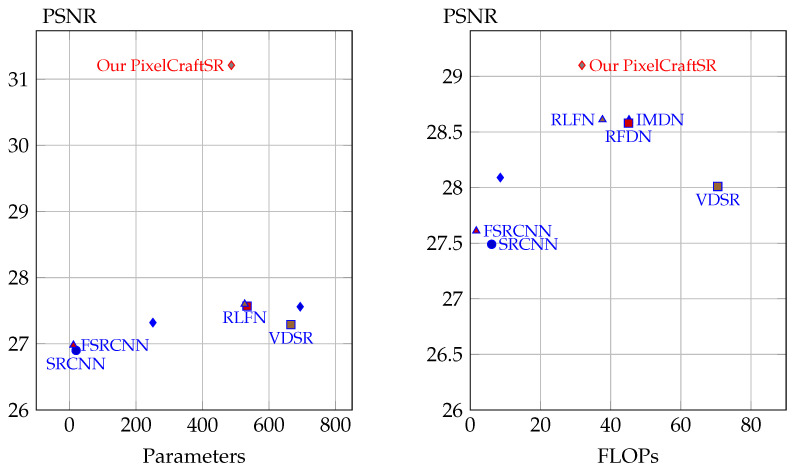
Comparison of the proposed method with state-of-the-art SR methods. Despite achieving state-of-the-art performance in quantitative evaluation, our method is computationally efficient.

**Figure 2:**
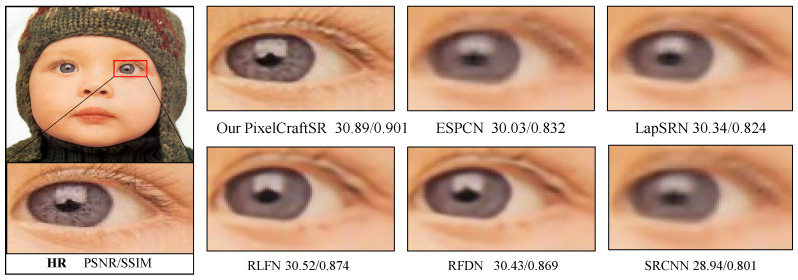
Quantitative comparison of SR at a scale factor of 4. It can be seen that the proposed method can produce salient details without any visually disturbing artifacts.

In response to the above challenges, we propose an alternative reinforcement-learning (RL)-based approach for making sequential decisions on images while gaining knowledge from their actions. Although RL-based methods are known to offer optimal solutions in different domains [24,25,26], they have not been investigated for vision tasks and image reconstruction. Consequently, we propose a pixel-wise multi-agent-based RL approach called PixelCraftSR, which can deploy a predefined set of actions and maintain a certain policy to generate a higher-quality final output image. By utilizing this approach, we can address the adaptability to diverse inputs and solve the issue that requires considerable training data. By incorporating a mix of deep-learning-based and traditional image-enhancement techniques in the action set, RL agents can leverage the strengths of both approaches, potentially leading to more effective results. In general, iterative actions and their corresponding response in the form of improved images than previously support the interpretability of the learning process. Figure 1 demonstrates that the proposed PixelcraftSR achieves a better trade-off between the peak signal-to-noise ratio (PSNR) and parameters compared with recent state-of-the-art approaches for ESR tasks. Additionally, a visual comparison is shown in Figure 2.

The contributions of this study can be summarized as follows:

- We propose PixelCraftSR, an RL-based method for ESR to deploy pixel-wise agents that adhere to the asynchronous advantage actor–critic (A3C) policy. This approach significantly improves the construction of super-resolution images while using considerably fewer parameters than existing ESR methods. To the best of our knowledge, this is the first approach to utilize RL in the ESR task.
- To create PixelCraftSR, we propose a novel action set that can be deployed at the pixel level. This action set is composed of three deep-learning-based ESR methods and four traditional image-enhancement techniques, collectively forming an effective ensemble strategy for SISR.
- Within our proposed action set, we introduced modifications to the SRCNN to enhance its performance further by increasing its depth and elevating channel-wise attention within the network.
- In addition, we deployed our model on the Jetson Nano Orin platform to evaluate its efficiency. Our approach demonstrated significantly faster and real-time output performance, highlighting its practical applicability for real-world scenarios.

The remainder of the paper is organized as follows: The following section provides an overview of our proposed RL-based SR method. Section 3 outlines the experimental setup and results. In Section 4, we offer our concluding remarks for the paper.

## 2. Related Work

The related works of this paper consist of two distinct components: the first part provides an overview of the deep-learning-based ESR approaches, while the second part covers reinforcement-learning-based image processing methods.

### 2.1. Deep-Learning-Based Efficient Super-Resolution

In the domain of single-image SR based on ESR tasks, a critical tradeoff exists between image quality and computational complexity [27]. Initially, the SRCNN [17] was introduced using a deep-learning approach. Although this approach is much more cost-effective, it has limited effectiveness for extreme upscaling, is vulnerable to artifacts, and lacks flexibility for diverse image characteristics. To address this issue, Kim et al. [18] proposed an architecture called VDSR, which increases the layer depth of the SRCNN to 20 layers. The authors performed Skip connections, which are used to mitigate the vanishing gradient problem associated with deep networks. This approach performed better than the SRCNN approach but still failed to produce significant results owing to training data dependency and susceptibility to overfitting when the training dataset was small or lacked diversity. In addition, the VDSR approach utilizes a fixed receptive field—considered a limitation of the architecture. Dong et al. proposed a network called FSRCNN [19] that utilizes transpose convolution for upsampling layers, which enables an efficient post-upsampling SR approach. This approach performed faster than other existing networks but failed to produce better visual outputs. In addition to the approaches mentioned above, Shi et al. [20] introduced ESPCN, a methodology that uses sub-pixel convolutional upscaling for SR image creation. This method involves restructuring the feature maps to improve resolution, and one noticeable element is the introduction of a sub-pixel l convolution layer. Although ESPCN has demonstrated excellent performance in providing high-quality results with computational efficiency in SISR applications, it may introduce artifacts such as ringing, halos, or other unnatural features into upscaled images. In addition, the LapSRN [28] approach utilizes a Laplacian pyramid framework to construct HR images. The authors attempted to improve LapSRN’s resolution using multiscale representations and information. This method aids in determining fine details as well as the overall quality of the SR images. However, this method failed to address the noise sensitivity issue because the network captured a high level of noise from the input, and the SR output exhibited noise amplification. However, the authors of IMDN [29] made notable advancements, surpassing the capabilities of pre-existing networks. IMDN introduces a simplified information multi-distillation network. This network comprises cascading information multi-distillation blocks that systematically extract hierarchical characteristics using an information distillation process. Later, the authors of RFDN [23] improved the IMDN by incorporating residual feature distillation links. While addressing time complexity, these models tend to create artifacts or blurriness in SR images. Recently, the authors of the RLFN [1] proposed a structure that leverages the idea of learning residual local features through three convolutional layers. Nevertheless, training this network poses significant challenges, mainly because of its recurrent nature, which makes it particularly susceptible to overfitting. A summary is presented in Table 1.

### 2.2. Reinforcement-Learning Based Image Super Resolution

Reinforcement learning (RL) is a machine-learning approach employed to train agents to generate decision sequences. It is frequently used in applications such as gaming, robotics, and autonomous systems [25,29,34]. Most deep-learning-based image-processing tasks require large amounts of data to train models. Acquiring substantial volumes of data poses significant challenges in certain domains. In addition, these methods failed to address robustness to noise and variations in the input data. Using reinforcement learning, learning from limited data becomes possible, leading to increased robustness against noise and variations in the input data. Vassilo et al. introduced a method that employed multiple GANs at both the pixel and patch levels [35]. Although this approach has demonstrated superior results in SR tasks, it remains impractical for ESR tasks owing to its high memory complexity and time-consuming nature.

## 3. Proposed Reinforcement-Learning-Based Super Resolution Method

**Figure 3:**
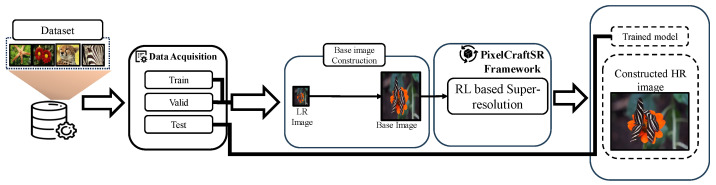
Overview of the proposed framework. We incorporated RL with lightweight deep networks to perform SR efficiently. We denoted our proposed method as PixelCraftSR.

In this section, we present the novel PixelCraftSR, which combines ESR networks with an RL framework, thus enabling the transfer of knowledge representations from ESR models to our proposed RL framework. Such a novel learning strategy helps our pixel-level decision method to construct superior SR images. Figure 3 presents an overview of the proposed framework.

### 3.1. Base Image Construction

In the proposed method, bicubic up-sampled LR images were initially input into PixelCraftSR. This process involves the construction of a foundational image using cost-effective interpolation techniques. We further investigated these interpolation methods by experimenting with bilinear interpolation, bicubic interpolation, and nearest-neighbor methods [36]. Notably, we consistently observed that bicubic interpolation yielded superior results compared to other interpolation methods. This approach facilitates our RL agents in rapidly attaining HR pixel values by providing an initial pixel value obtained through bicubic interpolation. The transition from bicubic pixel values to HR values is notably efficient and expedites convergence. Consequently, the focus shifted from the direct convergence of the LR pixel values to the HR values. This strategy has the added benefit of reducing the model parameter count. Equation (1) describes the procedure. In this context, $I_{t}$ represents the resultant base image, *B* denotes the function employed for bicubic image upsampling, and $I_{\mathrm{LR}}$ refers to the LR image.

(1) $$I_{t}=B\left(I_{\mathrm{LR}}\right)$$

Following the creation of the base image, it is introduced into our novel RL framework with the purpose of engendering SR on the given image.

### 3.2. Pixel-Wise A3C-Based Agent for PixelCraftSR

The A3C algorithm, a key improvement in deep RL, incorporates the actor–critic technique [35], in which an actor-network initiates actions comparable to a policy and a critic-network analyzes its usefulness as a value network—critical for complex decision-making. A3C’s unique asynchronous training, which involves parallel agent instances, runs independently, speeds up learning, and adds variations to the training trajectory [34].

**Figure 4:**
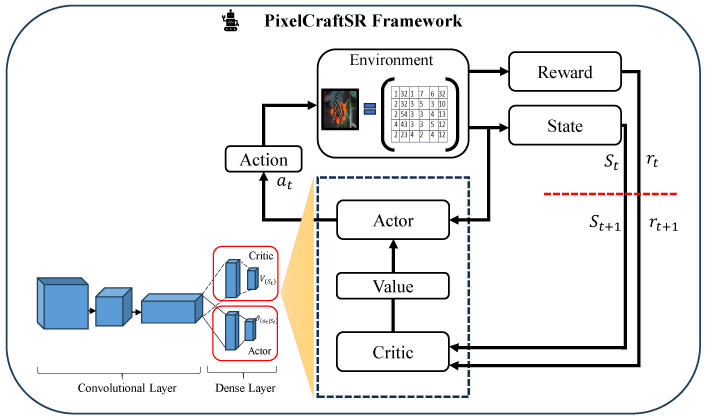
Overview of the proposed pixel-wise A3C algorithm-based SR workflow.

In the context of a pixel-wise agent deployment strategy for SR tasks, the actor–critic framework is utilized to optimize the quality of reconstructed HR pixels. For each pixel location, the Actor dynamically selects an action, $a_{t}$, based on the observed state $s_{t}$, which may include contextual features from the input image and past actions. The selected action directly influences the reconstruction of the corresponding HR pixel. Simultaneously, the Critic estimates the value function, $V(s_{t})$, which evaluates the expected long-term reward from the current state and helps refine the Actor’s policy through backpropagation. This interaction continues iteratively across all pixels. The entire workflow, including state transitions, action selection, value estimation, and reward feedback, is illustrated in the updated Figure 4, which explicitly visualizes the relationship between the Actor, Critic, and pixel-wise decision making process.

In our task, the Bellman equation, which incorporates the difference in mean squared error (MSE) values between successive states, guided the updates in the actor–critic methods. The policy gradient approach was used to update the actors.

(2) $$\nabla_{\theta }J=\frac{1}{B}\sum_{t=1}^{T}\nabla_{\theta }\log\pi_{\theta }\left(a_{t}|s_{t}\right)\cdot A_{\theta }\left(s_{t},a_{t}\right)$$

In Equation (2), the policy π with respect to its parameters θ provides guidance for enhancing the performance of the Actor. $\nabla_{\theta }\log\pi_{\theta }\left(a_{t}|s_{t}\right)$ calculates the gradient corresponding to the log probability of action selection $a_{t}$ at the state of $s_{t}$, and $A_{\theta }\left(s_{t},a_{t}\right)$ represent the advantage function, which captures the difference in quality between the built SR image and the Critic’s evaluation. The total time steps *T* and normalization by batch size *B* ensure that the policy variables are updated in a comprehensive and consistent manner.

(3) $$L=\frac{1}{B}\sum_{t=1}^{T}{\left(y_{t}-V_{\omega }\left(s_{t}\right)\right)}^{2}$$

Subsequently, the Critic’s parameters are updated to minimize the MSE loss. Equation (3) computes the squared difference between the expected value and the actual value $V_{\omega }\left(s_{t}\right)$ and the target value $y_{t}$ for each time step. The loss of the algorithm, which is aimed to be reduced throughout the training period, is denoted by *L*. These value updates ensure the iterative refinement of the agent’s policy and the accurate assessment of actions, ultimately contributing to the construction of high-quality SR images.

### 3.3. PixelCraftSR Action Set

In the proposed method, our pixel-wise agents employ a predefined action set, which includes the following actions mentioned in Table 2: increasing the pixel value by adding 1 to the corresponding pixel value in the base image or the image constructed from previous timesteps, decreasing the pixel value by subtracting 1 from the corresponding pixel value in the base image or the image constructed from previous timesteps, taking no action for that timestep, applying ESPCN at the pixel level, applying VDSR at the pixel level, applying our proposed novel SRCNN at the pixel level, and enhancing sharpness by increasing the pixel value corresponding to the base image or the image constructed from previous timesteps.

### 3.4. Modified SRCNN as an Action

**Figure 5:**
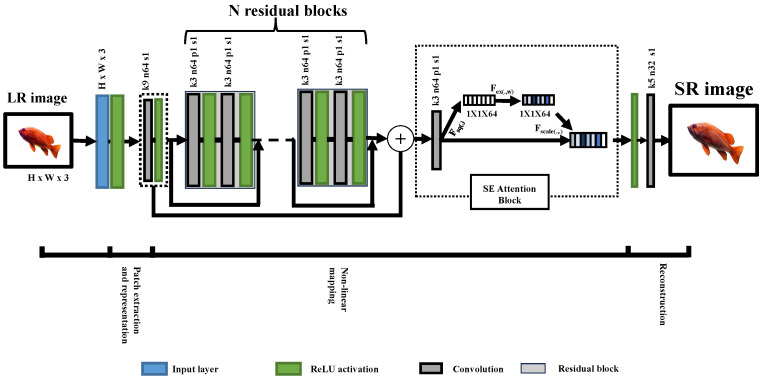
Network structure of our Modified SRCNN as an action. In the diagram, H represents the height, W represents the width, k represents the kernel size, n represents the number of channels, N represents the constant number, p represents padding, s represents stride, $F_{sq(.)}$ represents the squeeze function, $F_{ex()}$ represents the excitation function, and $F_{scale()}$ represents the scale-up function.

In the proposed RL-based approach, the primary goal is to achieve parameter efficiency while constructing promising quality images. When selecting deep-learning networks as actions for the action set, SRCNN is a more efficient choice regarding parameters. However, its limitation lies in its simple three-layer structure, which hinders its ability to effectively capture deep features effectively [37]. Consequently, in response to this challenge, we decided to modify and enhance the SRCNN architecture to capture deep features while maintaining a lower parameter count compared with other existing networks. To achieve this enhancement, we introduced an action designed to improve the SRCNN network architecture. This action involves incorporating multiple residual blocks and integrating squeeze and excitation (SE) attention blocks [38] into a non-linear mapping layer. These modifications allow the network to capture deeper features and recalibrate the significance of individual channels while generating the SR images. The proposed Modified SRCNN structure is illustrated in Figure 5. Based on this, we categorized our network into three layers: extraction and representation, non-linear mapping, and reconstruction layers.

**Patch extraction and representation layer:** 

Initially, the network receives the input of the LR image. This layer extracts the tiny overlapping patches. Subsequently, the extracted patches are represented as feature vectors. This procedure entails transforming each patch into a numerical representation such that the network’s future levels can be utilized. Equation (4) represents the patch extraction and representation layers formulae.

(4) $$F_{0}\left(I_{lr}\right)=max\left(0,W_{0}*I_{lr}+B_{0}\right)$$

Here, $W_{0}$ shows $n_{0}$ dimensional filters with sizes of *c* × $f_{0}$ × $f_{0}$ and $B_{0}$ biases, where *c* is the number of input channels of the $I_{lr}$ and $f_{0}$ × $f_{0}$ is the size of the kernel.

Non-linear mapping layer:

The output of the preceding layer, which consists of a convolutional layer followed by a rectified linear unit (ReLU) activation layer, represents a discrete block within the network architecture. After incorporating *n* number of residual blocks, the resulting output concatenates the output value with initial patch extraction and representation layer output. Subsequently, these values undergo a squeeze-and-excitation (SE) block operation to impart channel-wise significance to the features. In SE blocks, $F_{sq(.)}$ entails the computation of each channel’s global average pooled value. Subsequently, the excitation operation $F_{ex()}$ transforms the global information into channel-wise excitation weights [39]. Finally, $F_{scale()}$ scale the input feature map with the calculated channel-wise excitation weights.

The mathematical representation of the convolutional layer with rectified linear unit (ReLU) activation and the subsequent operation involving the residual and SE blocks is given by:

(5) $$F_{1}\left(F_{0}\right)=\max\left(0,W_{1}*F_{0}+B_{1}\right)$$

(6) $$F_{2}\left(F_{1}\right)=F_{SE}\left(F_{1}+F_{\mathrm{resblock}}\left(F_{1}\right)\right)$$

where extracted features are input for the $F_{1}$ function to map with $n_{2}$ dimensional filters and feed the next layer, where the concatenation of $F_{1}$ and $F_{resblock}$ is the input for SE the attention block.

Reconstruction layer:

In the case of Modified SRCNN action, the reconstruction layer is critical in translating the acquired characteristics into an HR image.

(7) $$F_{3}\left(F_{2}\right)=W_{2}*\left(max\left(0,F_{2}\right)+B_{2}\right)$$

where $F_{3}$ becomes a flattened form of a vector and $W_{2}*(max\left(0,F_{2}\right)+B_{2}$ represents the linear transformation applied to the activated features, which come as a reconstructed SR image.

### 3.5. Reward Function

A reward is a feedback value that an RL agent receives from its environment after performing an activity in a specific state [35]. The reward indicates how well the agent is progressing toward its goals. In our proposed PixelCraftSR, pixel-wise agents select actions based on $\sum_{i=0}^{j}\pi$. Here, *j* is denoted as the total number of agents. This approach is designed to efficiently and effectively construct SR images. The action selection procedure for each pixel-wise agent is expressed by Equation (8).

(8) $$a_{t}=\arg\max_{a}\left[\sum_{i=0}^{n}\left(\left|\right|H_{i}-P_{i}^{\left(t-1\right)}{\left|\right|}^{2}-\left|\right|H_{i}-P_{i}^{\left(t\right)}{\left|\right|}^{2}\right)\right]$$

At each time step, the reward function compares the output and prior HR images. In this equation, the reward associated with each time step within the *n*-timestep window is represented by the reward of each pixel at a specific time step. The action $a_{t}$ is selected to maximize the cumulative reward over that window. $P_{i}^{\left(t\right)}$ is the image from the previous time step and $P_{i}^{\left(t-1\right)}$ corresponds to the HR image. This equation illustrates the alteration in squared error between individual pixels and their targets following a specific action. When the agent selects an action that enhances the state, it results in a positive reward. Conversely, if the action deteriorates the state, the reward becomes negative. Our objective is to maximize the overall reward in Equation (8) by minimizing the squared error between each state and the HR image. This optimization encourages the output image to replicate the HR image.

### 3.6. Loss Function

Several prior studies have focused on adding perceptual loss to the feature space. Rather than analyzing the difference between the ground truth and generated images pixel-by-pixel, inaccuracy was measured within the feature space [35]. This enables the network to generate images with feature representations similar to those of the ground-truth images. In line with this, our suggested strategy stresses pixel-wise fidelity, with the VGG loss serving as the loss function. This loss function allows us to directly address and optimize the pixel-level accuracy, which contributes to the overall image quality improvement in our SR model. Initially, Sajadi et al. [40] derived feature representations by feeding HR and SR images through a pretrained implementation of the VGG-19 network. This involves extracting informative features from images using learned network parameters. The formula for VGG loss is as follows:

(9) $$\mathrm{VGG}\;\mathrm{Loss}=\frac{1}{N}\sum_{i=1}^{N}{\left(\phi \left(H_{i}\right)-\phi \left(P_{i}\right)\right)}^{2}$$

In this context, *N* refers to the total count of elements within the feature space. $H_{i}$ and $P_{i}$ represent the feature representations of the i-th element in the HR and SR images. The function ϕ indicates the feature extraction from the ’conv2_2’ layer of the VGG-19 network.

## 4. Experiments and Results

Initially, we constructed the training dataset, then we conducted the experiments. The experiments and results are presented in two main sections. First, the experiments and results of the Modified SRCNN are presented. Then, we discuss the experiments and results of applying our proposed PixelCraftSR framework, which leverages the Modified SRCNN, to our PixelCraftSR action set.

### 4.1. Dataset Preparation

We created a training dataset by combining the T91 [30], General100 [19], and BSD200 [41] datasets, resulting in 391 images. We validated the proposed method using the Set14 [42] dataset, and we tested the performance of the model using benchmark datasets such as Urban100 [43], BSDS100 [41], and Set5 [44]. The rationale for using this specific training dataset, rather than well-known large datasets such as DIV2K or Celeb, is to train a generalized model with a smaller dataset. This approach is practical because obtaining large datasets can be challenging in certain fields. Although DIV2K is a standard dataset with 800 natural images, our combined dataset consists of 391 images from T91, General100, and BSD200. These datasets were chosen owing to their diversity and representativeness across various image characteristics.

### 4.2. Modified SRCNN Action

In the context of our proposed Modified SRCNN (MSR-CNN), we employed the Adam optimizer to optimize the model and meticulously fine-tune its hyperparameters to ensure optimal performance. The training process spanned 100 epochs, with a batch size of 128. This rigorous training regimen, executed over 2 h 13 min, allowed us to refine the model parameters and effectively enhance its capabilities.

In this study, we systematically increased the number of residual blocks, denoted as *N*, starting from one. According to the results, it is evident that architectures with *N*values up to three exhibit an underfitting trend. However, when N=4, the training PSNR surpasses the validation (Set14) PSNR and structural similarity index (SSIM), providing better PSNR and SSIM values compared to other configurations with up to 4 residual blocks. We decided to add just one more residual block and conducted further experiments, observing a noticeable difference compared to other *N* values. We refrained from adding more residual blocks because our primary goal is to reduce the overall model parameters. The results are demonstrated in Table 3. Despite consistently delivering superior PSNR values compared to the VDSR approach, our MSRCNN, equipped with 5 residual blocks, falls short in terms of SSIM.

**Figure 6:**
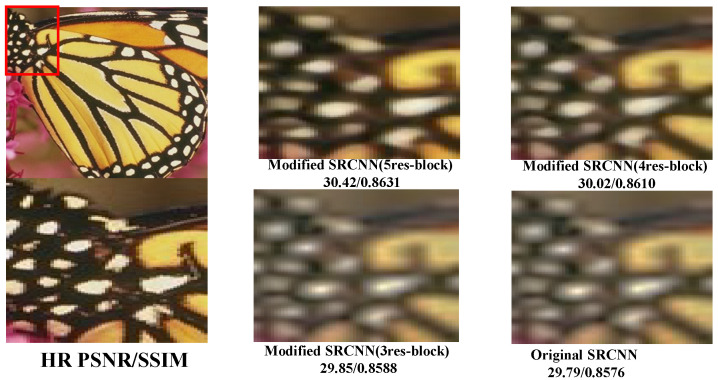
Qualitative performance analysis of Modified SRCNN architectures, illustrating enhanced efficacy of the 5 residual blocks configuration over the 4 and 3 residual block variants, and the original SRCNN.

In addition, Figure 6 demonstrates that our Modified SRCNN (5 residual blocks) architecture performs better than the configurations with 4 and 3 residual blocks, as well as the original SRCNN architecture.

### 4.3. Comparison of PixelCraftSR with SOTA Methods

#### 4.3.1. Quantitative Evaluation

We adopted a pragmatic approach in constructing the proposed PixelCraftSR, considering computational speed and memory constraints. Specifically, we conducted training on random image crops, each with dimensions of Y×Y, where we set the value of *Y* to 60. Those images were initially subjected to Gaussian blurring and downsampled based on the desired scale factor, a crucial step in the model training process. During this procedure, we employed scale factors of 2 and 4. We conducted training for the PixelCraftSR throughout 10,000 steps utilizing VGG loss, leveraging the computational power of the RTX 3050 GPU. This extensive training process allowed us to achieve optimal performance for our task. The results are demonstrated in Table 4.

**Figure 7:**
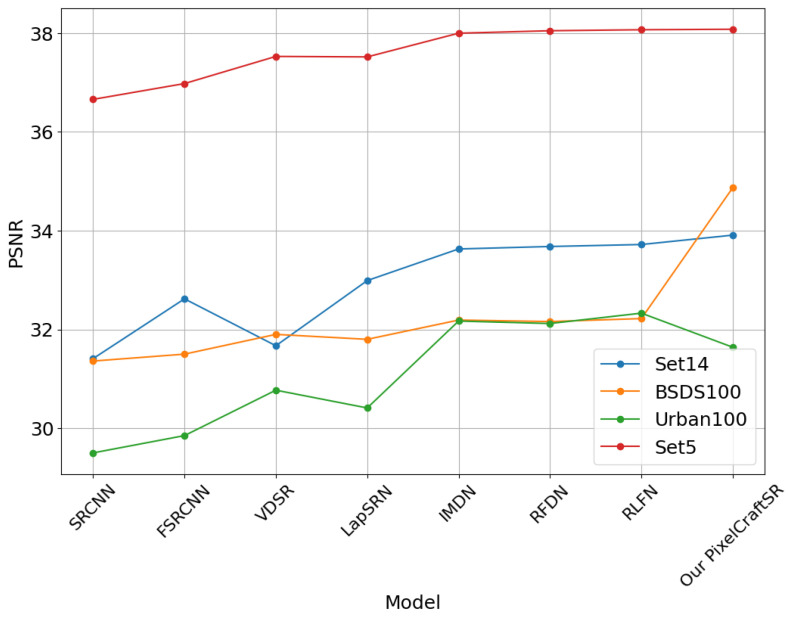
PSNR comparison for different datasets (Scaling Factor x2).

**Figure 8:**
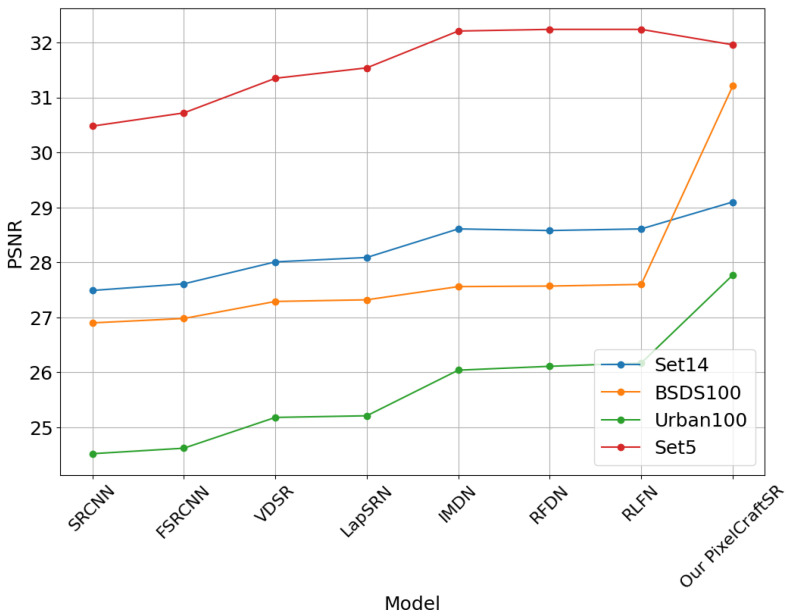
PSNRcomparison for different datasets (Scaling Factor x4).

**Figure 9:**
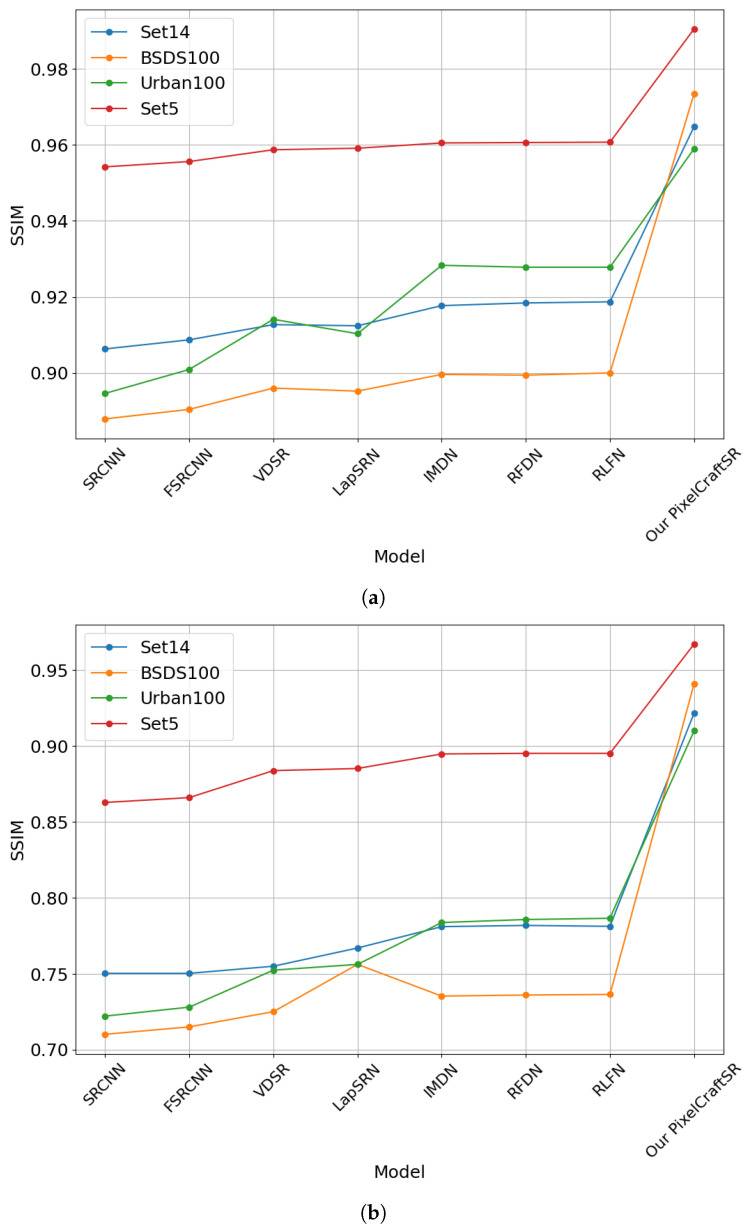
(**a**) SSIM comparison for different datasets (Scaling Factor x2). (**b**) SSIM comparison for different datasets (Scaling Factor x4).

Our proposed PixelCraftSR consistently achieves superior SSIM values across various datasets and scale factors. It also demonstrates efficiency by utilizing fewer parameters than most other methods, except for FSRCNN, LapSRN, and SRCNN. On the x2 Urban100 dataset, PixelCraftSR ranks fourth in PSNR, yet it excels in SSIM across all datasets. In Set5, our model secures the second-best PSNR, while achieving the highest SSIM value. Figure 7, Figure 8 and Figure 9 demonstrate that our proposed PixelCraftSR exhibits generalized performance across four benchmark datasets, measured in terms of SSIM and PSNR, across varying scale factors. PixelCraftSR not only outperforms most current state-of-the-art t models, including RLFN and RFDN, in terms of quality but also delivers a significant improvement in inference time.

#### 4.3.2. Qualitative Evaluation

**Figure 10:**
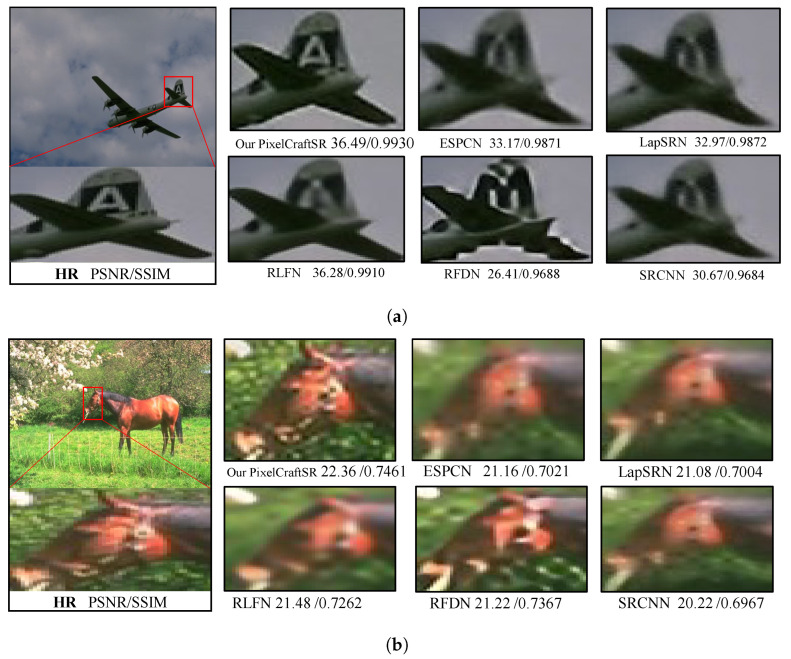
Qualitative comparisons between the proposed PixelCraftSR and state-of-the-art (SOTA) methods. (**a**) The x2 scaling factor with the image BSDS100-3096.png. (**b**) The x4 scaling factor with the image BSDS100-291000.png.

In Figure 10, we present the visual results for the SR output of our PixelCraftSR, along with the results from other state-of-the-art approaches at x2 and x4 scales. PixelCraftSR consistently outperforms the competing methods, exhibiting higher PSNR and SSIM values. Furthermore, our model uniquely reproduces finer characteristics, a distinction not observed in the results of other models.

### 4.4. Inference Analysis

To evaluate the efficiency of our model for real-world usage, we deployed our method on numerous hardware platforms, including an Nvidia GPU, X64 CPU (Santa Clara, CA, USA), and arm-based edge platforms (e.g., Jetson Orin). We evaluated our model using three image dimensions for the scaling factors x2 and x4. Table 5 lists the performance of the proposed method on numerous hardware platforms. It should be noted that we deployed and evaluated our model without performing any model optimization such as quantization and pruning. Despite model compression, the proposed method illustrates a satisfactory inference speed among all platforms. Additionally, our model can run seamlessly on edge devices, even in low-power modes (e.g., MAX-Q). This efficient and fast inference speed further confirms the practicability of the proposed method for real-world applications.

### 4.5. Ablation Study

#### 4.5.1. Modifying Action Set

**Figure 11:**
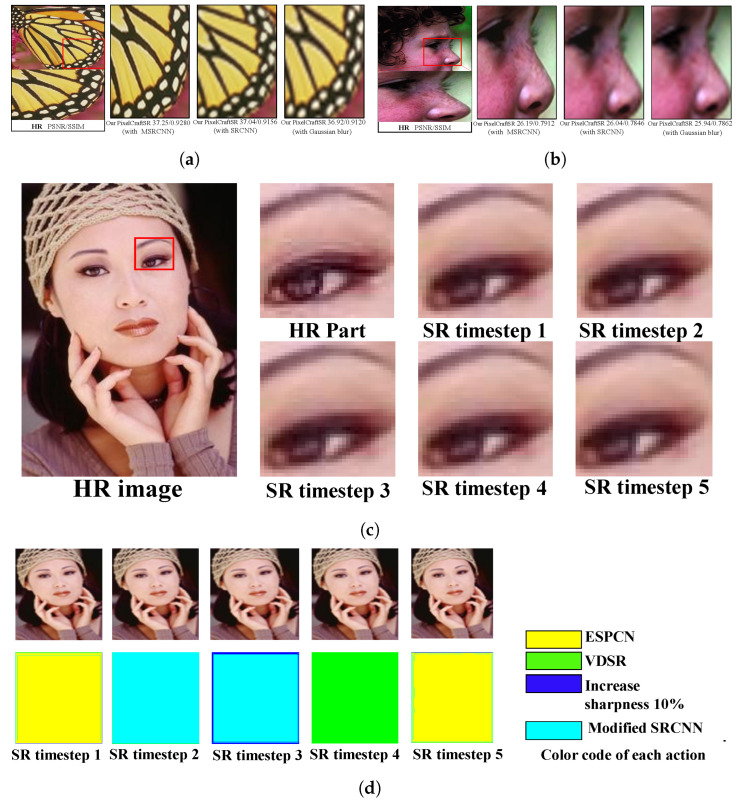
(**a**,**b**) Comparison of SR results using modified action sets of PixelCraftSR on Set5 dataset with different scale factors. (**c**,**d**) Visualization of PixelCraftSR’s progressive reconstruction and pixel-wise action selection behavior across time steps. (**a**) SR result comparison from the Set5 dataset img_003_SRF_2_LR.png with a scale factor of 2. (**b**) SR result comparison from the Set5 dataset img_004_SRF_4_LR.png with a scale factor of 4. (**c**) Progressive super-resolution of scale factor x2 for Set5 img_005_SRF_4_LR.png. Includes original HR and timestep-generated PixelCraftSR outputs. (**d**) Progressive action selection visualization for the same image, with color-coded actions.

To assess the effectiveness of the proposed PixelCraftSR, various experiments are conducted by modifying the action set of the proposed approach. Instead of using our proposed Modified SRCNN, we replaced the original SRCNN model and conducted the experiment. In contrast, we introduced an action, i.e., applying a Gaussian filter with σ=0.5 [25] to our PixelCraftSR. In comparison to the other two approaches, our PixelCraftSR demonstrated superior performance. The visual representation is illustrated in Figure 11 with the x2 and x4 scaling factors.

#### 4.5.2. Analysing PixelCraftSR Performance for Each Timestep

In the proposed PixelCraftSR framework, the SR output is generated in five time steps by applying pixel-wise actions dictated by the policy. Figure 11c visually presents output images for each time step, along with the HR part. This visualization distinctly demonstrates the gradual enhancement of the LR image at each time step, indicating significant improvement by the fifth step.

Figure 11d illustrates the pixel-wise action selection for the respective images across the five time steps, with each action represented by a unique color code. In the first time step, most central pixels employ the ESPCN, whereas border pixels use the VDSR approach. During the second time step, the center pixels utilize MSRCNN, and the border pixels continue to use VDSR. In the third time step, sharpening is applied to the border pixels, whereas the fourth time step considers the full application of the VDSR action to all pixels. In the final time step, VDSR is again applied to the border pixels, whereas ESPCN is used at the center.

## 5. Conclusions

In this study, we presented PixelCraftSR, a novel multi-agent RL-based approach to SISR, specifically designed for resource-constrained edge devices. By leveraging an iterative multi-agent process, our method significantly reduces model complexity, achieving only 2.81 G FLOPs and 487 K parameters while maintaining superior performance in PSNR and SSIM compared to state-of-the-art lightweight models such as RFDN and RLFN.

One of the main strengths of our approach lies in its ability to generalize effectively even when trained on smaller datasets, making it suitable for real-world scenarios where large scale datasets are unavailable. Moreover, the step-wise nature of our reinforcement-learning framework enables a more interpretable learning process than conventional black-box deep-learning models.

In terms of quantitative results, PixelCraftSR achieves 33.21/0.9418 (PSNR/SSIM) on Set14 and 31.21/0.9412 on BSDS100 for the ×4 scale. It also demonstrates faster inference, with an average runtime of 0.003 s per image, making it highly suitable for real-time applications on edge devices.

Despite these advantages, our approach is not without limitations. The RL-based framework introduces challenges related to training stability and convergence, often requiring the careful tuning of hyperparameters. Additionally, while our model performs well on standard benchmark datasets, further validation across a broader range of complex, high-resolution real-world images is necessary to confirm its robustness and adaptability beyond controlled environments.

For researchers and practitioners, PixelCraftSR provides a practical and interpretable framework for achieving high-quality SR under computational constraints. Its balance of efficiency, accuracy, and transparency makes it an appealing option for deployment on mobile and embedded platforms, while also offering a promising direction for future research in interpretable and efficient image super-resolution.

In conclusion, PixelCraftSR offers a compelling lightweight solution for SR tasks, combining interpretability, strong performance, and deployment efficiency, positioning it as a valuable contribution to both academic and practical advancements in edge-based image enhancement.

## Figures and Tables

**Table 1:** Limitation analysis of efficient SR approaches.

Approach	Published Venue	Training Dataset	Limitations
SRCNN [17]	TPAMI	T91 dataset [30] + ILSVRC 2013 ImageNet [31]	Struggles to generalize well to diverse image types and scales due to its reliance on fixed-size patches and lack of explicit understanding of image content.
FSRCNN [19]	ECCV	General100 [19] + T91 dataset [30]	Suffers from reduced performance on very large upscaling factors due to its reliance on iterative upsampling and convolutional layers.
ESPCN [20]	CVPR	T91 dataset [30] + BSD500 [32]	Exhibits artifacts and blurring in SR outputs due to the sub-pixel convolutional layer’s limited ability to reconstruct fine details.
IMDN [29]	Proc. ACM Inter. Conf. on MM	DIV2K [33]	The iterative approach increases computational complexity and training times, potentially affecting the quality of the output due to challenges in preserving fine details and avoiding artifacts, especially for extreme upscaling factors.
LapSRN [28]	CVPR	T91 dataset [30] + BSD500 [32]	The Laplacian pyramid structure potentially limits its scalability to high-quality output.
RFDN [23]	ECCV	DIV2K [33]	Increased computational complexity and potential challenges in capturing diverse image features—affecting the overall quality and generalization capability of the model.
RLFN [1]	CVPRW	DIV2K [33]	Local features lead to limitations in capturing global context and intricate details, potentially resulting in less accurate reconstruction of complex image structures and textures.

**Table 2:** Description of our proposed action set for PixelCraftSR framework.

No of Action	Action
1	Pixel value −1
2	Does nothing on that timestep
3	Pixel value +1
4	ESPCN [20]
5	VDSR [18]
6	Modified SRCNN; refer to Figure 5
7	Increase sharpness by 10%

**Table 3:** Comparing our proposed Modified SRCNN variants with other models: Training peak signal-to-noise ratio (PSNR), Validation PSNR, floating-point operations (FLOPs), and structural similarity index (SSIM) for various scaling factors.

Method	Train PSNR/SSIM	Validation PSNR/SSIM		FLOPs (G)	Param. (k)
		**Set14**	**Set5**		
SRCNN [17]	31.05/0.8923	31.41/0.9063	36.66/0.9542	6.10	20
MSRCNN4	31.86/0.9001	31.75/0.9072	36.72/0.9552	18.30	316
MSRCNN5	32.16/0.9051	31.88/0.9098	37.58/0.9567	24.45	389
VDSR [18]	32.00/0.9102	31.67/0.9127	37.53/0.9587	70.50	666

**Table 4:** Comparative analysis of SR models on four benchmark datasets: Parameters, floating-point operations (FLOPs), peak signal-to-noise ratio (PSNR), and structural similarity index (SSIM) for various scaling factors.

Scale	Model	Time (s)	Params (k)	FLOPs (G)	Set14 (PSNR/SSIM)	BSDS100 (PSNR/SSIM)	Urban100 (PSNR/SSIM)	Set5 (PSNR/SSIM)
x2	SRCNN [17]	**0.01**	20	6.10	31.41/0.9063	31.36/0.8879	29.50/0.8946	36.66/0.9542
	FSRCNN [19]	**0.01**	**12**	**1.72**	32.62/0.9087	31.50/0.8904	29.85/0.9009	36.98/0.9556
	VDSR [18]	0.23	666	70.50	31.67/0.9127	31.90/0.8960	30.77/0.9141	37.53/0.9587
	LapSRN [28]	0.71	251	8.57	32.99/0.9124	31.80/0.8952	30.41/0.9103	37.52/0.9591
	IMDN [29]	0.85	694	45.23	33.63/0.9177	32.19/0.8996	32.17/0.9283	38.00/0.9605
	RFDN [23]	0.05	534	37.67	33.68/0.9184	32.16/0.8994	32.12/0.9278	38.05/0.9606
	RLFN [1]	0.03	527	35.45	33.72/0.9187	32.22/0.9000	**32.33**/0.9278	38.07/0.9607
	Our PixelCraftSR	0.02	487	31.82	**33.91/0.9648**	**34.87/0.9735**	31.64/**0.9590**	**38.08/0.9905**
x4	SRCNN [17]	**0.01**	20	6.10	27.49/0.7503	26.90/0.7101	24.52/0.7221	30.48/0.8628
	FSRCNN [19]	**0.01**	**12**	**1.72**	27.61/0.7503	26.98/0.7150	24.62/0.7280	30.72/0.8660
	VDSR [18]	0.23	666	70.50	28.01/0.7550	27.29/0.7250	25.18/0.7524	31.35/0.8838
	LapSRN [28]	0.82	502	8.57	28.09/0.7670	27.32/0.7562	25.21/0.7562	31.54/0.8852
	IMDN [29]	0.91	715	45.23	28.61/0.7811	27.56/0.7353	26.04/0.7838	32.21/0.8948
	RFDN [23]	0.05	550	45.10	28.58/0.7819	27.57/0.7360	26.11/0.7858	32.24/0.8952
	RLFN [1]	0.04	543	37.67	28.61/0.7813	27.60/0.7364	26.17/0.7866	**32.24**/0.8952
	Our PixelCraftSR	0.03	487	31.82	**29.10/0.9218**	**31.21/0.9412**	**27.77/0.9100**	31.96/**0.9673**

**Table 5:** Performance Evaluation of PixelCraftSR on CPU, GPU, and Jetson Orin Platforms.

Scale	Input Dimension	FLOPs (G)	Inference Speed (s)			
			**CPU**	**GPU**	**MAX-Q**	**MAX-N**
x2	256×256	31.82	0.42	0.02	0.05	0.05
	512×512	74.58	0.87	0.04	0.09	0.12
	1024×1024	149.17	1.92	0.07	0.32	0.44
x4	256×256	31.82	0.43	0.03	0.05	0.06
	512×512	74.58	0.96	0.04	0.09	0.12
	1024×1024	149.17	1.89	0.07	0.33	0.44

## Data Availability

The datasets used and/or analyzed during the current study are available from the corresponding author upon reasonable request.
